# Combined Porous-Monolithic TiNi Materials Surface-Modified with Electron Beam for New-Generation Rib Endoprostheses

**DOI:** 10.3390/jfb14050277

**Published:** 2023-05-15

**Authors:** Anastasiia V. Shabalina, Sergey G. Anikeev, Sergei A. Kulinich, Nadezhda V. Artyukhova, Vitaly A. Vlasov, Maria I. Kaftaranova, Valentina N. Hodorenko, Evgeny V. Yakovlev, Evgeny A. Pesterev, Anna V. Lukyanenko, Mikhail N. Volochaev, Sofiya Pakholkina, Oibek Mamazakirov, Victor V. Stolyarov, Anatolii V. Mokshin, Victor E. Gunther

**Affiliations:** 1 Laboratory of Medical Materials Science, Tomsk State University, 634050 Tomsk, Russia; 2 Institute of Physics, Kazan Federal University, 420008 Kazan, Russia; 3 Research Institute of Science and Technology, Tokai University, Hiratsuka 259-1292, Kanagawa, Japan; 4 Research School of High-Energy Physics, National Research Tomsk Polytechnic University, 634050 Tomsk, Russia; 5 Tomsk Scientific Center, Siberian Branch of Russian Academy of Sciences, 634055 Tomsk, Russia; 6 Kirensky Institute of Physics, Federal Research Center, KSC Siberian Branch Russian Academy of Science, 660036 Krasnoyarsk, Russia; 7 School of Engineering Physics and Radio Electronics, Siberian Federal University, 660041 Krasnoyarsk, Russia; 8 Department of Morphology and Physiology of the Medical Institute, Surgut State University, 628403 Surgut, Russia

**Keywords:** TiNi, rib endoprostheses, porous coating, powder metallurgy, high-current pulsed electron beam, structure, surface modification, electrochemical corrosion, biocompatibility

## Abstract

TiNi alloys are very widely used materials in implant fabrication. When applied in rib replacement, they are required to be manufactured as combined porous-monolithic structures, ideally with a thin, porous part well-adhered to its monolithic substrate. Additionally, good biocompatibility, high corrosion resistance and mechanical durability are also highly demanded. So far, all these parameters have not been achieved in one material, which is why an active search in the field is still underway. In the present study, we prepared new porous-monolithic TiNi materials by sintering a TiNi powder (0–100 µm) on monolithic TiNi plates, followed by surface modification with a high-current pulsed electron beam. The obtained materials were evaluated by a set of surface and phase analysis methods, after which their corrosion resistance and biocompatibility (hemolysis, cytotoxicity, and cell viability) were evaluated. Finally, cell growth tests were conducted. In comparison with flat TiNi monoliths, the newly developed materials were found to have better corrosion resistance, also demonstrating good biocompatibility and potential for cell growth on their surface. Thus, the newly developed porous-on-monolith TiNi materials with different surface porosity and morphology showed promise as potential new-generation implants for use in rib endoprostheses.

## 1. Introduction

TiNi and its alloys are widely used as materials in medicine due to their unique and attractive properties [1,2,3,4,5,6,7,8,9]. Devices designed on their use have successfully proved themselves in a wide range of areas, such as the development of tools [10,11], cardiology [12,13,14,15], orthopedics [16,17], surgery [18,19,20], ophthalmology [21,22], and so on. Both monolithic and porous materials based on TiNi are extensively used to fabricate implantable structures. Owing to the implementation of thermoelastic martensitic transformations, monolithic materials exhibit the very good mechanical characteristics required for implanted constructions while having quite poor adhesion of tissue cells to their smooth surfaces [23,24,25]. At the same time, porous TiNi materials, while being more compatible with porous bone tissues and demonstrating better cell attachment and growth, do not exhibit satisfactory mechanical performances. Therefore, combining both monolithic and porous TiNi structures into one material for implantation seems to be a very promising approach. In such a material, its monolithic part is necessary to improve the deformation and strength characteristics under alternating loads, whereas the porous part increases the survival rate of the implanted structure due to its better attachment to surrounding biological tissues of a varied nature (connective, cartilaginous, bone).

Porous monolithic or composite TiNi materials are already widely used for the creation of endoprostheses targeting different goals, such as the replacement of resected fragments of ribs [26], elimination of defects in the lower jaw [27], dental implantation [28], etc. All the above-mentioned implantable devices and structures based on TiNi typically have a similar composite structure that combines a monolithic TiNi plate and a relatively thick, porous TiNi part obtained by sintering or by self-propagating high-temperature synthesis (SHS). Endoprostheses of the lower jaw and dental implants have relatively massive porous parts as they do not experience bending and should survive one-dimensional mechanical loads. At the same time, since rib implants are subjected to bending, their porous TiNi part should be as thin as possible in order to prevent a decrease in the deformation resource of their underlaying monolithic substrate.

In addition, all endoprostheses of the ribs known so far experience the problem of the eruption of their porous part, the latter problem manifesting itself over time as such prostheses are exploited for long periods of time [29,30,31]. This problem is known to be exacerbated by two groups of factors: (i) physiological (high deformation loads in the process of respiratory activity and as a result of the everyday physical activity of a person), and (ii) technological (difficulties in making a high-quality contact at the boundary of the monolithic and porous parts).

From the technological point of view, the boundary between the porous and monolithic parts in the artificial ribs requires special attention. Initially, such endoprostheses were produced by mechanically connecting monolithic TiNi plates and porous SHS-produced plates using titanium rivets or by fastening them together with thin threads. However, this type of connection is not reliable since the porous plates can potentially crack or break up around the rivets. As a next step, a more complex preparation approach was proposed in which the monolithic plate was loaded in a quartz tube for SHS, surrounded by a Ti-Ni charge, so that the SHS proceeded on its surface and around it. After that, the rib endoprosthesis was machined from an as-prepared cylindrical billet. The disadvantage of this preparation method is its high consumption of materials, labor and time (with a high percentage of waste generated during prosthesis formation). Additionally, the percentage of defective items is known to increase when the SHS temperature regime is violated during their manufacturing violated. In the latter case, traces of pure nickel can remain at the boundary with the monolithic part, which is inacceptable for implants. To avoid this issue, the starting temperature of the SHS stage was increased. However, this resulted in much larger surface pores and interpore bridges [26,27,32]. In addition, because of the technological limitations of the SHS method, the porous part of the finished endoprosthesis is often made to be as thick as up to 3–5 mm, which also limits the deformation resource of the implant [26,27,32]. Thus, up to date, the technology for fabrication of high-performance TiNi materials for artificial ribs is still far from being reliable, cost-efficient, and well reproducible.

To address the above-mentioned challenges and prepare TiNi composite materials with a thin, porous layer on a monolithic substrate, albeit with high adhesion between them, we proposed properly sintering selected TiNi powders on monolithic plates with a subsequent modification of the formed composite structure [26,27,32]. After surface modification with a low-energy high-current pulsed electron beam (HCPEB), the fabricated materials were shown to have a relatively thin TiNi-based porous layer on monolith TiNi plates, while the porosity and adhesion of the porous layer was able to be tuned through processing parameters [33,34,35,36]. As-fabricated materials can meet the requirements for rib endoprostheses.

In our previous research, we demonstrated that sintering of TiNi powder with sizes 100–200 µm, followed by HCPEB treatment, allowed us to produce composite TiNi surfaces with roughness parameters *R*_a_ and *R*_z_ within the range of 60–80 and 280–360 µm, respectively [33,34,35,36]. For such relatively large powder particles, the sintering mode used was limited to the sintering temperature of 1200 °C. As a result, the depth of modified layer was found to be 5–20 μm. Thus, the thickness of the porous layer, and especially its adhesion strength to the monolithic substrate, still remained issues for further research.

In the course of the further development of composite TiNi materials consisting of porous layer on monolith substrate, the present study focused on producing such materials by sintering and surface HCPEB processing smaller TiNi powders with initially spongy morphologies. Owing to the more developed macrostructure of such powder particles and the terrace-like features on their surface, the use of such smaller TiNi powders was expected to increase the specific surface area of materials based on them.

Thus, the present study aimed to develop new biocompatible porous-monolithic materials based on TiNi for use as new-generation rib endoprostheses. With this overall goal in mind, in this study, we: (i) produced such composite TiNi materials by means of the surface sintering of small-sized TiNi powders, followed by further HCPEB treatment; (ii) studied the effect of manufacturing parameters on a product’s surface roughness and morphology; (iii) studied corrosion resistance of prepared composite materials and compared it with similar materials produced using a larger TiNi powder as precursor, as well as with initial TiNi monolith; and (iv) evaluated biocompatibility of produced composite TiNi materials by observing cell growth on their surface.

## 2. Materials and Methods

### 2.1. Material Preparation

To prepare samples with combined porous-monolithic structure, monolithic plates and TiNi powder, prepared via the calcium hydride approach [37,38,39,40,41], were used.

Plates of Ni (N1, purity of 99.90%) and spongy titanium (TG-90, purity of 99.94%), both purchased from “Ural Metals”, Kamensk-Uralsky, Russia, were remelted in an ISV-0.004-PI M1 (Petra, Ufa, Russia) induction furnace filled with inert argon gas (99.99% “Cryogenmash-Gas”, Tomsk, Russia) to obtain the initial monolithic TiNi plate. The equiatomic ratio of the melt was achieved with the help of a GH-200 balance (A&D, Tokyo, Japan). After solidification, the resulting ingots (cylindrical, 300 mm long and 25 mm in diameter) were processed in a DOU-80 rolling mill (DOU, Moscow, Russia) until thickness of 2 mm and the width of 15 mm was reached. Annealing temperatures of 800–1000 °C were used in a SUOL 0.4.4/12 tube furnace (Tula-Term, Tula, Russia) in order to relieve stresses after rolling. For etching, the material was immersed in an acidic solution (3H_2_O + 2HNO_3_ + 1HF) for 2–3 s. Both acids, nitric (65%) and hydrofluoric (45%), were chemically pure in grade and were purchased from SIGMATEK (Khimki, Russia). After etching, the material was washed using water and alcohol. The as-prepared TiNi plate was also characterized for the purpose of comparison and denoted as TiNi-Pl (see Table 1).

To prepare the porous part of the materials, TiNi powder of grade PV-N55T45 (Polema, Tula, Russia) was used. Powder particles of a desired size range (0–100 µm) were obtained by grinding larger fractions and screening with calibration sieves. Then, the powder was placed on a monolithic plate for sintering. The powder-on-monolith blanks were placed in a quartz capsule and sintered at a temperature of 1200 °C for 15 min in an electric vacuum furnace SNVE-1.31/16-I4 (VARP, Moscow, Russia). Thus, sample SP was obtained (Table 1). To evaluate the temperature influence on the monolithic part of the material, sample TiNi-Pl. was heat-treated under the same conditions, giving rise to sample TiNi-Pl./t (see Table 1).

Replicas of sample SP were then taken in order to prepare samples SP-20 and SP-30, as the latter samples were subjected to additional treatment by an electron beam in different modes performed in a RITMSP station (Microsplav Inc., Tomsk, Russia), as previously described by Markov and co-authors [42]. A high current (up to 25 kA) and 30 pulses with a pulse duration of 2–4 s with beam energy of 20 and 30 keV were used to fabricate samples SP-20 and SP-30, respectively. The beam diameter was up to 80 mm, with energy density being around 3 J/cm^2^ for the SP-20 sample, and 6 J/cm^2^ for the SP-30 sample. In the present study, in addition to the series of small-pored (SP) materials, we also prepared their large-pored (LP) counterparts, the latter materials being obtained under the same conditions but with the use of larger TiNi powders, as was previously reported elsewhere [36] (Table 1).

### 2.2. Sample Characterization

The granulometric composition of initial powder was studied using a laser particle size analyzer, Analysette 22 NanoTec plus (Fritsch, Idar-Oberstein, Germany). Scanning electron microscopy (SEM) studies were performed on a Quanta 200 3D system (FEI Company, Hillsboro, OR, USA). Surface roughness parameters, such as maximum deviation *R*_z_ and average roughness *R*_a_, were found using optical profilometry (MNP-1 tool from Technological Design Institute of Scientific Instrument Engineering, Siberian Branch of RAS, Russia) and the instrument’s original software. X-ray diffraction (XRD) patterns of the samples were recorded on an XRD 6000 diffractometer (Shimadzu, Kyoto, Japan) within the range of 2θ = 20–100° at a sampling rate of 0.02°/s using CuKα radiation. The PDF-4 database and Powder Cell 2.5 software, equipped with pseudo-Voigt profile functions, were used in phase analysis.

### 2.3. Electrochemical Corrosion Study

Electrochemical measurements were performed on an R-40X potentiostat-galvanostat (Electrochemical Instruments, Chernogolovka, Russia). We used an E-7SF three-electrode cell filled with saline (0.9% NaCl) with a graphite auxiliary electrode and an Ag/AgCl reference electrode (4.2 M KCl). The test material was pressed against the current collector and contacted with the electrolyte through a window in the bottom of the cell that was 10 mm in diameter. The potential varied from −1 to +1 V, with a sweep rate of 3 mV/s. Tafel plots were obtained from the recorded data, while values of corrosion potential *E*_corr_ and corrosion current *I_corr_* were found graphically.

To calculate the corrosion rate, it was necessary to determine the surface area that came into contact with the electrolyte during the experiment. The studied samples had different roughness; this is the factor that determines the specific surface area of the material. Since in this study the main task was to compare materials with each other, it was decided to normalize the surfaces of materials according to the values of their roughness parameters. For this, we used a *Ti* plate as a flat standard, thus taking its roughness parameter as 1. For all the other materials tested, their *R_a_* value was divided by that of the *Ti* plate (Formula (1)). The resulting coefficient (*f^n^*) was put into the formula for calculating the corrosion rate instead of the surface area (Formula (2)). Thus, the obtained relative corrosion rate for the materials ($C_{corr}^{*}$) allowed us to compare the materials with each other.

(1) $$f^{n}=Ra_{n}/Ra_{Ti}\;$$

(2) $$C_{corr}^{*}=3.27\times 10^{-3}I_{corr}EW/\rho f^{n},$$

where the roughness values *R_a_* were measured separately for each material; *EW* is the equivalent weight of the material, in this case equal to 26.36 (dimensionless); *ρ* is the density of the material. A more detailed description of all formula members along with their dimensions can be found in ASTM G102 (Standard Practice for Calculation of Corrosion Rates and Related Information from Electrochemical Measurements, Section 3.2).

### 2.4. Biocompatibility and Cytotoxicity Experiments

#### 2.4.1. Hemolysis Study

The human blood of a healthy volunteer was used. It was mixed with a sodium citrate solution (3.8 wt.%). Then, it was diluted with saline 9:1. Samples (TiNi-Pl. and LP) were immersed in a standard test tube containing 10 mL of saline preincubated at 37 °C for 30 min. After that, 0.2 mL of diluted blood was added to this standard tube and incubated for 60 min at 37 °C. Normal saline used as a negative control, and deionized water as a positive control, were treated similarly. Then, all tubes were centrifuged for 5 min at 3000 rpm in a 2–7 Cyto centrifuge (Sigma Laborzentrifugen, Osterode am Harz, Germany) and the supernatant was carefully transferred to a cuvette for spectroscopic analysis. Hemolysis was calculated using an ultraviolet spectrophotometer (Picon, Uniplan, Russia) using (Formula (3)):

(3) $$Hemolysis=\left(OD_{test}-OD_{neg.}\right)÷\left(OD_{pos.}-OD_{neg.}\right)\times 100\%$$

where *OD* is the optical density, “*test*” denotes tested material with blood, while “*neg*.” and “*pos.*” are used for negative and positive controls, respectively.

#### 2.4.2. Cytotoxicity Study

The MTT assay was used to evaluate the cytotoxicity of TiNi-Pl and LP samples.

MCF-7 cells were cultured in Dulbecco’s modified Eagle’s medium (DMEM), 10% fetal bovine serum (FBS), antibiotics (100 U/mL penicillin and 100 mg/mL streptomycin) and 2 mM L-glutamine at 37 °C in a humidified room with an atmosphere containing 5% of CO_2_.

Tests for cytotoxicity were performed by direct contact. Control groups included the use of DMEM as a negative control and 0.64% phenolic DMEM as a positive control. Cells were incubated in 12-well cell culture plates with media in each well with samples incubated for 24–72 h. After that, MTT was added to each well. Samples were incubated with MTT for 4 h at 37 °C; then, the plates were centrifuged for 10 min at 1500 rpm. The supernatant was carefully removed, and a solution of solubilized formazan (10% SDS in 0.01 M HCl) was added to each well. The spectrophotometric absorbance of the samples was measured at 540 nm on a Picon (Picon, Uniplan, Russia) with a reference wavelength of 630 nm.

#### 2.4.3. Cell Viability Study

The viability of human peripheral blood mononuclear cells was studied.

At first, 10 mL of blood was diluted 1:1 with PBS, layered on ficoll and centrifuged at 2000 rpm for 30 min. Thus, a layer containing mononuclear cells was obtained. Then, 5 mL of the buffy coat sample were washed twice with 10 mL of PBS, centrifuged at 1500 rpm for 5 min, and placed in a 12-well plate containing 2 × 10^6^ cells/mL of RPMI-1640 (Paneco, Moscow, Russia). We added to this 10% fetal bovine serum (Paneco, Moscow, Russia), antibiotics (100 units/mL penicillin and 100 mg/mL streptomycin) and 2 mM L-glutamine at 37 °C in a 5% CO_2_ atmosphere. The studied samples were placed in a 12-well plate and incubated for 24 and 72 h. The viability of this product was then determined using the trypan blue dye exclusion method.

#### 2.4.4. Cell Growth Study

All procedures involving animals were carefully carried out with strict adherence to the Declaration of Helsinki of 1975 and in accordance with the European Community’s Council Directive 86/609/EEC. The study protocol was officially approved (approval code number 20/1116/2017 of 5 May 2017) by the Bioethical Committee of Tomsk State University. Bone marrow stem cells of F1 CBA/j hybrid mice were used as cellular material. The femur was removed under sterile conditions, after which the bone marrow was washed out with a syringe into vials.

The cell concentration was adjusted to 10^7^ cells/mL of complete medium, after which they were seeded onto TiNi samples and placed in 50 mL plastic bottles (Corning, NY, USA). Cultivation took place in a medium that consisted of DMEM-F12 medium (Paneco, Moscow, Russia), 10% fetal calf serum (HyClone, USA), gentamicin 40 μg/mL (Paneco, Moscow, Russia), and glutamine 250 mg/mL (Paneco, Moscow, Russia).

Differentiation additives were used in the system with osteogenic differentiation: beta-glycerophosphate 3 mg/mL (Sigma-Aldrich, St. Louis, MO, USA) was used in combination with 0.15 mg/mL ascorbic acid (Sigma-Aldrich, St. Louis, MO, USA). Incubators with cells were kept at T = 37 °C and 100% humidity with a 5% CO_2_ concentration. Experimental samples were examined on days 1, 7, 10, and 17. Incubators were fixed for 1 h in 2.5% glutaraldehyde (Sigma-Aldrich, St. Louis, MO, USA), washed three times in PBS medium (15 min each), then fixed for 1 h in 1% osmium tetroxide (Sigma-Aldrich, St. Louis, MO, USA), washed 3 times in PBS and then dehydrated by passing through a series of ethanol solutions (30%, 50%, 70%, 90%, 100%) for 15 min each.

SEM and confocal laser scanning microscopy (CLSM on LSM 780 NLO, Zeiss, Germany) were used to observe the cells growing on the samples. The second method was performed for non-dried samples using suitable vital stains (acridine orange and ethidium bromide).

## 3. Results and Discussion

### 3.1. Structure and Composition of Combined Monolithic-Porous Material

#### 3.1.1. Monolithic Part of the Material

A two-roll rolling mill was used to obtain a TiNi monolithic plate after melting the ingot. This allowed us to produce 2 mm-thick plates from a cylindrical ingot. After being subjected to multiple rolling and intermediate annealing procedures, the as-prepared TiNi monolithic material was covered with a massive TiO_2_ layer up to 300 µm in thickness. The latter layer had a granular structure and it was constantly renewed during processing because, in the process of compression, its outer part cracked and fragmented. Bilateral deformation is known to lead to the formation of a longitudinally textured oxide layer which, in addition to titanium oxide, may also contain secondary elements (C, Si, Fe, etc.) that enter into it during thermo-mechanical treatments. Additionally, because surface oxides are known often to prevent the surface of a monolithic plate from being wetted by a melt during sintering [43], such materials should be cleaned from their massive oxide layer before covering their monolithic part with a porous powder layer.

**Figure 1:**
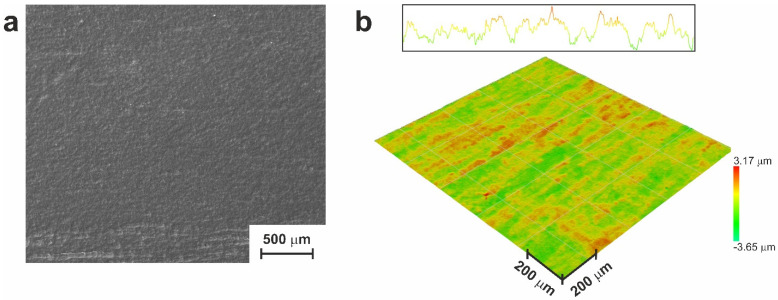
Surface morphology of chemically etched monolithic TiNi plate used for the further preparation of monolithic-porous material: (**a**) SEM image; (**b**) optical profilometry image.

For surface cleaning, chemical etching with an aqueous solution of nitric and hydrofluoric acids was used in the present study. Such a treatment removed the oxide layer, along with the TiNi and Ti_2_Ni compounds. Since TiNi is the main phase of the prepared monolithic material and particles of the secondary Ti_2_Ni phase have sizes in the range of 0.1–3 µm, the TiNi plate developed surface craters up to 3 µm in size after chemical pretreatment. Further etching led to the gradual disappearance of such craters, eventually resulting in the TiNi plate whose surface morphology is presented in Figure 1. The average roughness parameter of the material after chemical etching was *R*_a_ = 0.5 µm, with the maximum profile deviation *R*_z_ = 5.5 µm. This surface topography was considered appropriate for the further deposition of a porous layer through sintering TiNi powder and subjecting it to HCPEB processing.

#### 3.1.2. TiNi Powder Used in the Study

**Figure 2:**
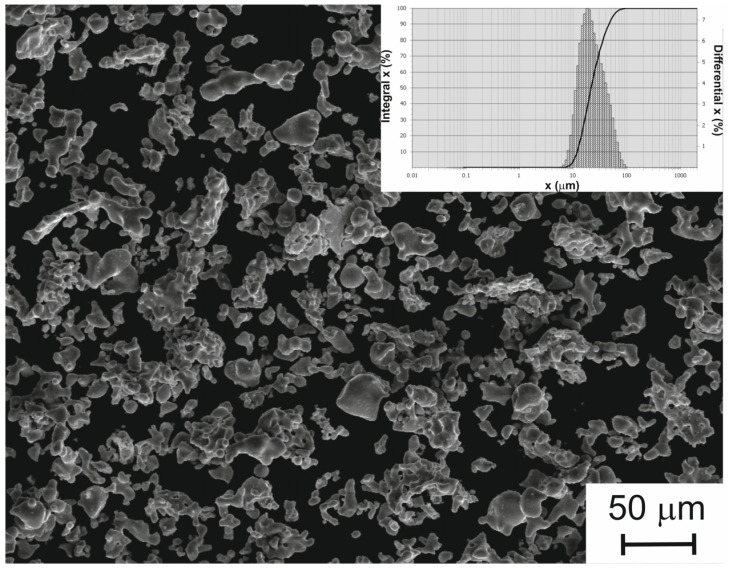
SEM image of smaller fraction of TiNi powder used to prepare porous TiNi layer. Inset: particle size distribution.

As mentioned in the Experimental section, the powder particles in the desired size range were obtained by grinding larger fractions of the same powder. TiNi powders obtained via calcium hydride reduction are known to have a spongy structure [37]. Their grinding therefore led to the destruction of individual large spongy particles and the formation of spongy and compact ones with drop-like and dumbbell-like shapes, as seen in Figure 2. The average particle size of the resulting powder was found to be approximately 25.5 µm and had a normal Gaussian distribution (Figure 2, Inset). The use of smaller particles (with sizes up to 100 μm) resulted in an increase in the specific surface area of resultant powder to 470 cm^2^/g (or 3050 cm^2^/cm^3^), while the use of a larger fraction with sizes 100–200 μm showed a lower value of 65 cm^2^/g (or 410 cm^2^/cm^3^).

Thus, the fabrication of a thin, porous TiNi alloy based on a powder with sizes below 100 µm may potentially permit to obtain a thin and more structurally developed surface layer on a monolithic TiNi substrate.

#### 3.1.3. Resultant Structure of TiNi Powder and Monolithic Parts and Their Subsequent Treatment

**Figure 3:**
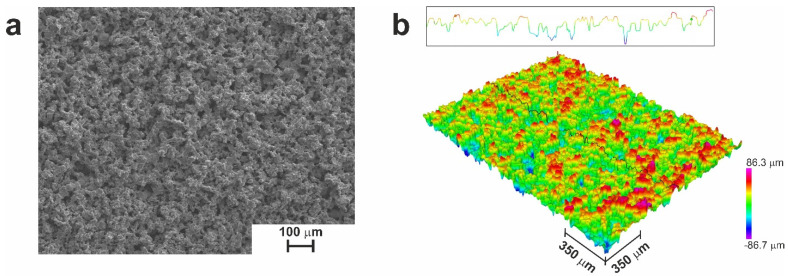
Surface morphology of sample SP: (**a**) SEM image; (**b**) optical profilometry image.

Sintering the above-mentioned TiNi powder on the surface of the preprepared monolithic TiNi plate at a temperature of 1200 °C led to a well-developed porous surface structure with *R*_a_ of 22 µm and *R*_z_ of 140 µm (Figure 3).

**Figure 4:**
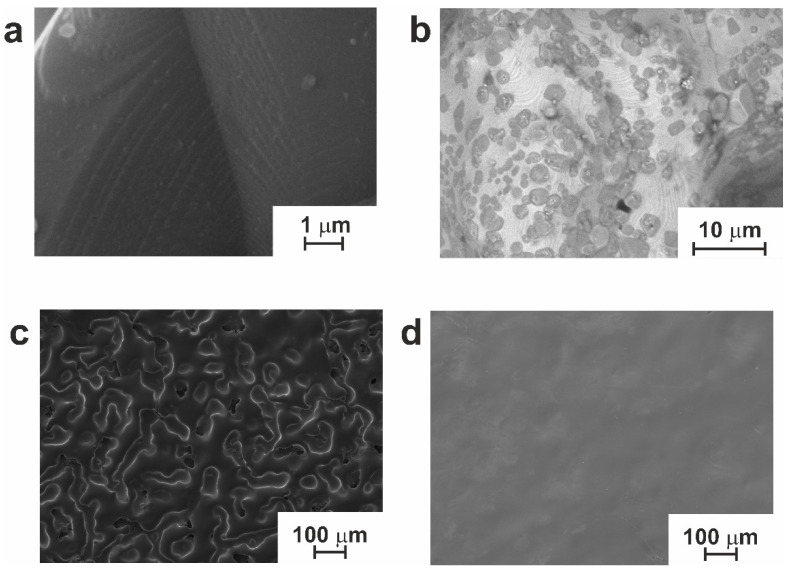
SEM images of the obtained materials: (**a**) terraces and (**b**) secondary phase particles on the surface of sample SP; (**c**) sample SP-20; (**d**) sample SP-30.

It can be seen that the surface of the manufactured material inherited the sponge-like structure of sintered powder particles. Additionally, specific terrace-like structures could be found on the surface of the TiNi phase of the material (Figure 4a). Additionally, during high-temperature processing, the inevitable interaction with carbon atoms from oil vapor (from the diffusion pump) occurred, with Ti_2_Ni secondary phase particles dissolving it to form the Ti_4_Ni_2_(O,C) oxycarbide phases seen in Figure 4b. According to report [2], the crystal lattice parameters of Ti_2_Ni and oxycarbide phases are close. Therefore, identification was carried out in this study based on EDX. Our previous experience revealed that Ti_4_Ni_2_(O,C) particles demonstrate more straightened interphase boundaries. Additionally, they reach 5–7 µm in size, while Ti_2_Ni phase particles are typically spherical and not larger than 2–3 µm.

Figure 4 also shows an SEM surface image of sample SP. This was prepared after treatment with the electron beam in modes 20 and 30. It can be seen in Figure 4c that sample SP-20 (prepared in mode 20) preserved the developed surface structure of the initial powder material. On the contrary, sample SP-30 exhibits an excessive degree of electron beam processing. This led to a surface structure consisting of concave and convex sections (Figure 4d).

Energy beam treatment is known to homogenize the surface of processed materials. Repeated exposure to a high-energy electron beam leads to the melting of surface layers that are up to several micrometers in thickness. With an increase in pulse number, the volume of forming liquid phase increases due to thermal heating. Over time, the latter liquid phase intermixes and partially evaporates, while rapid crystallization occurs during the intervals between irradiation pulses. Non-equilibrium phase transformations proceed in the surface layer, giving rise to ultrafine-grained or nanosized structures [44]. However, the presence in the surface layer of secondary-phase particles (e.g., Ti_2_Ni), oxycarbonitrides, or titanium carbide TiC was reported to lead to the formation of craters [45,46]. The repeated irradiation of the formed material induces the formation of new craters; thus, the overlapping and melting of such craters eventually leads to surface smoothening. This is why the surface morphology and surface roughness parameters of sample SP-30 were found to approach those of the original monolithic plate, as seen in Figure 4d and Table 2.

Thus, sintering followed by modification with electron beam permits to effectively control the macrostructure of the porous top layer formed on the surface of monolithic TiNi material.

#### 3.1.4. Composition of Obtained Materials

**Figure 5:**
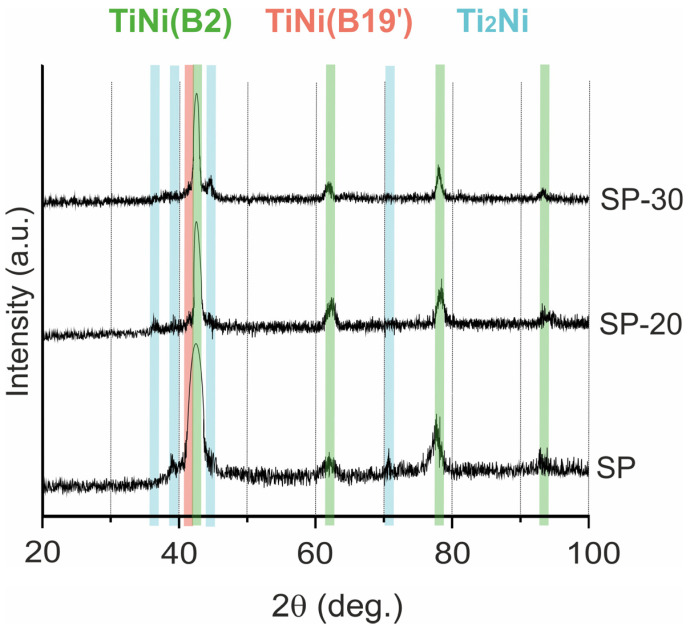
XRD patterns of the SP group materials.

The results of XRD analysis of the SP group samples are presented in Figure 5: the XRD patterns of all the samples demonstrate the matrix compound TiNi in austenitic (B2) and martensitic (B19′) states. The presence of second-phase particles Ti_2_Ni/Ti_4_Ni_2_(O,C) was also confirmed, and the calculated content of all detected phases is presented in Table 3. It can be seen that, with an increase in the energy of the electron beam impact, the volume fraction of the main TiNi phase increases while the volume fraction of secondary phases decreases. This finding is consistent with the studies of several other teams [47,48,49]. During electron beam treatment, desorption of light elements (O,C) from the surface and secondary recrystallization to a depth of up to 20 µm occurred. This led to the dissolution of second-phase particles in the irradiated part of the powder layer. Henceforth, as a result of electron beam treatment, the phase composition of the material’s top layer was homogenized.

### 3.2. Corrosion Study of the Materials

In the present study, in addition to corrosion performance of newly developed materials (materials of the SP series), for comparison we also tested the corrosion performance of counterpart materials prepared with larger TiNi particles (materials of the LP series) whose preparation was reported elsewhere [36]. This allowed us to reveal the effect of the particle size of TiNi powders on the corrosion resistance of potential rib implants manufactured on their basis (materials of series LP and SP).

**Figure 6:**
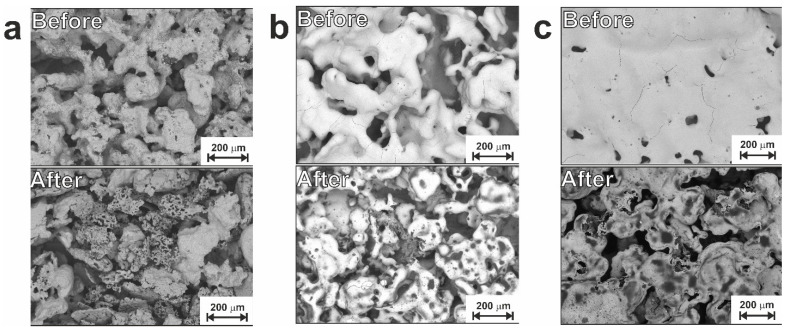
SEM images of the LP group samples before (upper row) and after (lower row) corrosion: (**a**) sample LP; (**b**) sample LP-20; (**c**) sample LP-30.

**Figure 7:**
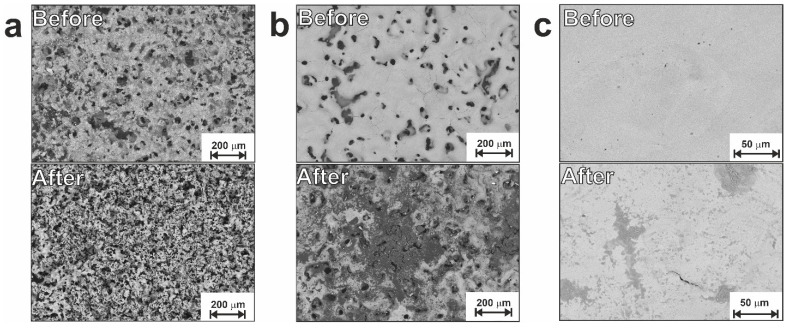
SEM images of the SP group samples before (upper row) and after (lower row) corrosion: (**a**) sample SP; (**b**) sample SP-20; (**c**) sample SP-30.

Figure 6, Figure 7 and Figures S1–S7 show microscopic images of samples from the LP and SP series taken before and after electrochemical corrosion tests. It can be seen that after polarization cycles, all materials undergo changes in the structure and composition of their surface. Based on the images obtained after corrosion (low rows in Figure 6 and Figure 7), one can assume uneven corrosion and local corrosion, including pitting and corrosion by spots.

**Figure 8:**
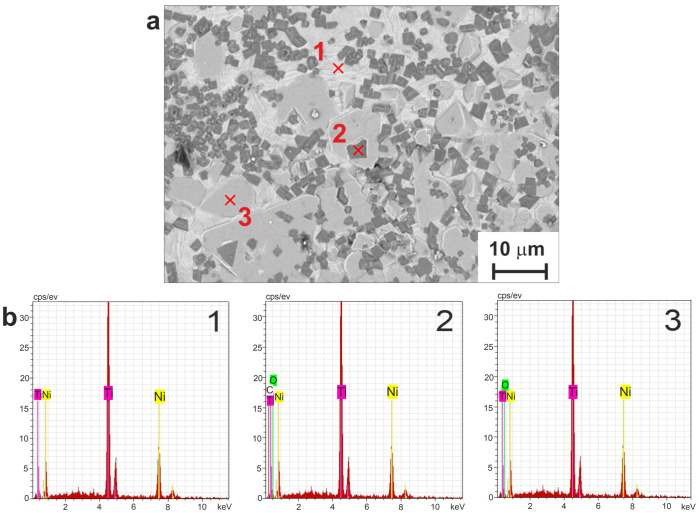
(**a**) SEM surface image of sample LP subjected to corrosion, with marked areas analyzed by EDX; (**b**) EDX spectra taken at points marked in panel (**a**).

Two corrosion processes are possible for the prepared combined porous-monolithic materials: (1) structural corrosion, which is associated with the initial structural inhomogeneity of the surface of the samples; (2) selective corrosion, which destroys only one component of the material or one of its structural components (for example, only protrusions or only the bottom of pores). Both these processes were observed for the LP and SP series samples, with corrosion products quite clearly seen on the surface in Figure 6 and Figure 7 (lower rows). Bulk particles located on the surface, after it has undergone corrosion, are of a particular interest. Hence, we applied EDX to various particles and found two distinct types exhibited in Figure 8. It can clearly be seen that while one contains C and O atoms, the other one shows only O as an impurity (compare spectra for points 1 and 2). Both types formed from the Ti_2_Ni phase and are generally defined as the Ti_4_Ni_2_(O,C) class.

**Figure 9:**
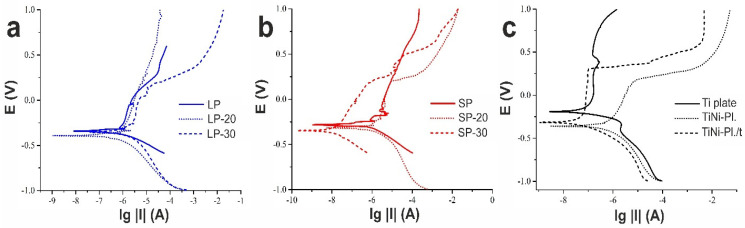
Tafel curves for materials of (**a**) LP group; (**b**) SP group; (**c**) Ti plate and TiNi plates.

The Tafel curves recorded during electrochemical corrosion tests for all studies materials (as well as for Ti and TiNi plates used as reference) are shown in Figure 9. The obtained data allowed us to graphically determine the values of corrosion current and corrosion potential which are all presented in Table 4. Since, in the present study, the real (electrochemical) surface area of the samples could not be determined, the corrosion rates of the materials could not be evaluated. Thus, for the sake of comparison, Table 4 presents values of $C_{corr}^{*}$ which were calculated taking into account samples’ surface roughness (See Section 2 for details).

**Figure 10:**
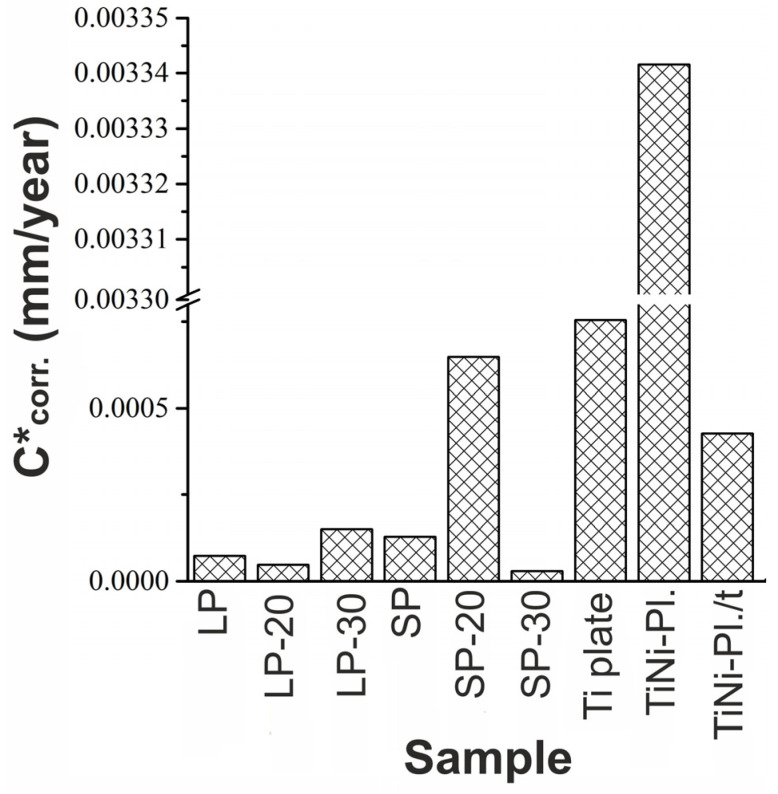
Corrosion rate values evaluated for all studied samples and their comparison with Ti plate, TiNi plate, and thermally treated TiNi plate. Surface roughness of all samples was taken into account.

It can be seen in Table 4 that the most positive value of the corrosion potential was demonstrated by the Ti plate. Thus, according to the values of corrosion potential, one can conclude that this material was the most resistant to corrosion when compared to the other samples. However, the most reliable way to assess the corrosion resistance of a material is to analyze its corrosion rate. Therefore, in Figure 10, a general histogram of calculated corrosion rates is presented.

Figure 10 shows that the highest corrosion rate was observed for the original TiNi plate. This was expected since the plate had a lot of surface defects which were potential sites for corrosion. After heat treatment, the corrosion rate of the same plate (TiNi-Pl./t) expectedly decreased. This occurred as annealing led to surface passivation through the growth of thicker surface oxide layers. Thus, thermal treatment increases the resistance of TiNi to corrosion in a biological environment.

In general, all the other tested materials demonstrated corrosion rates lower than that of the reference Ti plate. That is, their corrosion resistance in biological media was better than that of titanium (which is a standard material for numerous implants). Among the two-dimensional porous materials based on TiNi, the lowest corrosion rate was found for samples LP, LP-20, and SP-30 (see Figure 10).

An interesting point worth discussing is the anomalously increased corrosion rate seen in Figure 10 for sample SP-20, especially in comparison with samples SP and SP-30. Unlike the other samples, the powder-based layer on sample SP-20 was found to crack during polarization (see Figure S5c), which could have a crucial effect on the sample’s behavior. It can be assumed that cracks, alongside the freshly exposed surfaces associated with them, were the reason for and sites of higher-rate corrosion.

Thus, with a comprehensive consideration of corrosion parameters extracted from electrochemical measurements, we demonstrated that the newly developed materials, comprising porous and monolithic TiNi parts, both with and without additional modification with electron beam, exhibit increased corrosion resistance compared to monolithic TiNi alloy (see Figure 10). The obtained results will pave the road for further research in this direction aiming at finding the best optimal conditions for powder-based coating with highest possible corrosion resistance.

### 3.3. Biomedical Characterization

#### 3.3.1. Hemolysis and Cytotoxicity Study

Hemolysis and cytotoxicity were studied for both the TiNi plate and one of the newly developed materials based on a combination of monolithic and porous TiNi, namely sample LP. Standard tests for biocompatible materials showed that the hemolysis of erythrocytes was (1.98 ± 0.34)% and (1.65 ± 0.31)% for the samples TiNi-Pl. and LP, respectively. Thus, having the degree of hemolysis below 2%, the newly developed TiNi materials meet the standard requirements for biocompatible materials that come into contact with the circulatory system according to standard ISO 10993-4: 2020 [50,51].

The index of cytotoxicity studied using the MTT test during daily cultivation with MCF-7 line cells showed a decrease from (24 ± 2.2)% for TiNi-Pl, with no porous layer, to (13 ± 1.3)% for the sample LP. Thus, the newly developed TiNi materials with a porous surface were shown to reduce the cytotoxicity index by 11% when compared with a conventional flat TiNi plate.

The viability of human peripheral blood mononuclear cells was assessed during their long-term cultivation in the presence of sample LP. The experiment, carried out for up to 72 h, demonstrated an increase in the viability of human blood mononuclear cells, showing (80 ± 5)% on day two and (62 ± 4)% on day three. For comparison, sample TiNi-Pl showed similar values of (71 ± 5)% and (46 ± 4)% for day two and day three, respectively. Since the composition of both materials was similar, we conclude that the surface morphology affected the cellular biocompatibility observed. This implies that the modification of TiNi with a porous layer has long-term potential for cytocompatibility.

#### 3.3.2. Cell Growth Experiments

The test was carried out with cell cultures for different periods of cultivation (namely, 3, 7, 10, and 17 days) and using SEM and CLSM for visualization.

Figures S8 and S9 show the surface of materials after 3 days of cell growth. Separate cells, rather than agglomerates, were visible on the surface of both series LP and SP samples. Cells grown after one week are shown in Figures S10 and S11, where the number of cells is clearly larger than it was after 3 days. In general, at the initial stage (days 3–7), the best results in terms of cytocompatibility were demonstrated by the SP series samples, especially SP-20 (Figures S8–S11, Supplementary Materials). In general, both electron and confocal microscopy showed an increase in the number of cells on the surface of all small-pored samples when compared with their large-pored counterparts. This may be explained by the increased surface area of the SP samples and surface terrace-like structures, both of which apparently lead to better cell adhesion to such samples.

**Figure 11:**
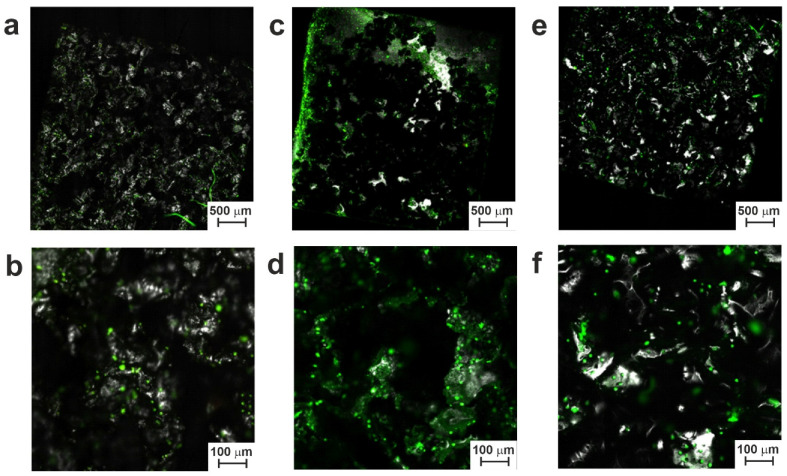
CLSM images of materials from the LP group after 17 days of cell growth: (**a**,**b**) sample LP; (**c**,**d**) sample LP-20; (**e**,**f**) sample LP-30.

**Figure 12:**
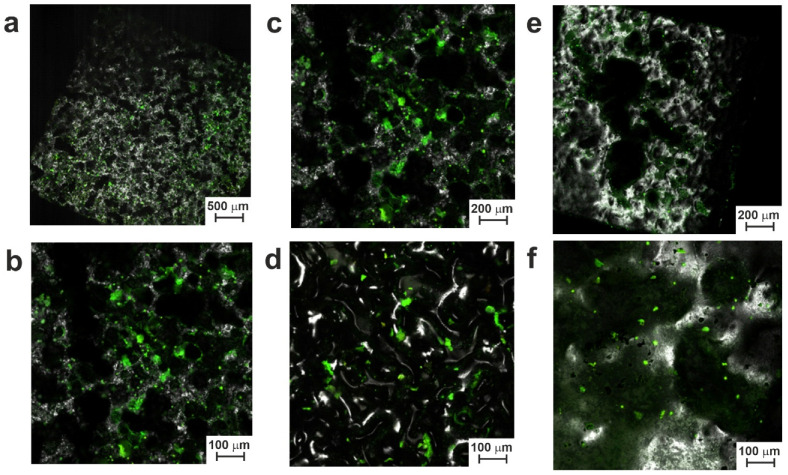
CLSM images of materials from the SP group after 17 days of cell growth: (**a**,**b**) sample SP; (**c**,**d**) sample SP-20; (**e**,**f**) sample SP-30.

As can be clearly seen in Figures S8–S11, during the initial periods of growth, cells were mainly located in local surface trenches and valleys that had formed along with surface hillocks as a result of TiNi fusion on a smooth TiNi substrate and the subsequent electron beam treatment of samples 20 and 30. This could be explained by the spontaneous sedimentation of cells in suspension and the ingress of more cells before their attachment to surface valleys. As the cell colonies grew further, they gradually populated the tops and hillocks of the surface (Figure 11, Figure 12, Figures S12 and S13, Supplementary Materials). This indicates that cell culture can successfully colonize and grow on the porous TiNi surfaces developed in this study as potential materials for implants.

It should be noted that while at initial stages, the SP samples demonstrated more colonies on their surface; after days 10–17, more cells and cell agglomerations were found on electron beam-treated samples from the LP group (see Figure 11, Figure 12, Figures S12 and S13, Supplementary Materials). This implies that surface morphology indeed played a crucial role in cell growth dynamics on the samples prepared in this study.

In addition to cells, adult tissue is known to contain an intercellular matrix with colloidal and fibrous components. After 10 days of cultivation, the population of bone marrow stromal cells was found to continue developing on the tested samples. By day 10, most of the cells were found to protrude the plasma membrane and form intercellular connections. Thus, Figure 11, Figure 12, Figures S12 and S13 exhibit the areas where growing cells accumulated on the tested surfaces. On day 17 of cell growth, an increase in the formation of the intercellular matrix was seen, which is known to happen when the required critical level mass is achieved by growing cells. At this point, the concentration gradient of growth and nutritional factors inside local surface valleys increases, and the cells gradually fill their initial locations, moving further and covering the nearby hillocks on the sample’s surface (Figure 11 and Figure 12).

The obtained results are in good agreement with study [52], where the dynamics of the interaction of cell populations with the surface of an alloy based on TiNi was considered. This study showed that implantable devices with a microroughness of 20–145 µm could successfully resist embolism. Textured surfaces are known to promote better adhesion and proliferation of cell population [53,54,55,56,57]. In our experiments, we observed a gradual increase in cell mass and extracellular matrix for both series, samples LP and SP. This indicates a positive contribution of the surface structure of the obtained materials to their biocompatibility.

Obviously, the small-pored samples with more developed surface structures (of the SP series) are more favorable for initial cell fixation on the material surface. However, the further growth of cell populations proceeds better on smoother surfaces (of the LP series). This can be explained by easier cell motion over short distances on a smoother surface in comparison with more energy-demanding expansion and growth on a rougher surface. Hence, the optimization of surface morphology is necessary to provide a TiNi material with a good balance between the initial adhesion of cells and their further spreading over the entire surface.

Thus, based on the obtained results, it can be concluded that the materials developed in the present study, with their fused surface structures based on TiNi powders, are biocompatible with mesenchymal cells of the bone marrow of CBA mice and, consequently, with their derivatives: bone, cartilage, and adipose tissues. At the same time, the use of different powder fractions and different parameters for electron beam processing during sample preparation permits to tune surface morphology and the way the produced material interacts with surrounding living tissues.

## 4. Conclusions

Aiming at developing new-generation materials for rib endoprostheses, this study reports on newly prepared TiNi materials combining thin, porous layers and monolithic plates as their substrates. The materials were obtained using a TiNi powder with relatively small (below 100 µm) particles which were sintered on monolithic TiNi plates, followed by their subsequent treatment with high-current pulsed electron beam (HCPEB). By tuning beam parameters, a series of porous monolithic TiNi materials with small-pored surface layers were prepared. For comparison, a similar series of large-pored materials was prepared by applying a similar TiNi powder with lager particles (100–200 µm).

Before HCPEB treatment, sintered small-pored materials demonstrated terrace-like features on their porous surface, while after irradiation with high-energy beam they exhibited homogenized surface structure and composition due to the dissolution of secondary phases. Expectedly, the surface of such materials was more porous and rougher than that of the initial TiNi monolith used as substrate.

Comparison with large-pored samples demonstrates that the use of TiNi powders with different sizes for the surface modification of monolithic substrate permits to tune surface morphology and roughness, which is important for manufacturing implants with tuned cell adhesion.

Electrochemical corrosion experiments, carried out on both small-pored and large-pored porous-on-monolith TiNi materials, demonstrated the improved corrosive resistance of such materials in comparison with the flat TiNi monolith used as their substrate. Thus, the corrosion resistance of powder-modified monolith TiNi materials tested in biological media was found to be good enough for their application as implants.

Hemolysis degree was found to be below 2%, implying that the newly developed TiNi materials meet the standard requirements for biocompatible materials in contact with the circulatory system. The cytotoxicity index was revealed to be lower by 11% in comparison with the initial flat TiNi monolith plate, and the viability test of human peripheral blood mononuclear cells showed that the modification of TiNi with a porous layer has a long-term potential for cytocompatibility.

Cell growth experiments carried out for both large-pored and small-pored porous monolithic materials showed their biocompatibility with mesenchymal cells of the bone marrow of CBA mice and, consequently, with their derivatives: bone, cartilage, and adipose tissues. During the test, cell spreading was observed to be dependent on surface morphology. This implies that the ability of the approach used to tune surface roughness and morphology is very useful for optimizing the surface of potential implant to maximize its compatibility with surrounding tissues.

Overall, the newly developed porous-on-monolith TiNi materials with different surface porosity and morphology show promise as potential new-generation implants for rib endoprostheses.

## Figures and Tables

**Table 1:** Samples’ nomenclature and details of their preparation.

Sample Name	Plate	Powder	Sintering	Energy Mode of HCPEB
TiNi-Pl.	TiNi	–	–	–
TiNi-Pl./t	TiNi	–	1200 °C for 15 min	–
SP	TiNi	0–100 µm	1200 °C for 15 min	–
SP-20	TiNi	0–100 µm	1200 °C for 15 min	20 keV
SP-30	TiNi	0–100 µm	1200 °C for 15 min	30 keV
LP	TiNi	100–200 µm	1200 °C for 15 min	–
LP-20	TiNi	100–200 µm	1200 °C for 15 min	20 keV
LP-30	TiNi	100–200 µm	1200 °C for 15 min	30 keV

**Table 2:** Roughness parameters of the initial monolithic TiNi plate and materials of the SP group.

Sample	*R*_a_ (µm)	*R*_z_ (µm)
TiNi-Pl.	0.5	5.5
SP	22	140
SP-20	18	90
SP-30	3	15

**Table 3:** Phase composition of the SP group samples according to XRD data.

Phase	Content in Samples (vol.%)		
	SP	SP-20	SP-30
TiNi (B2)	73	85	91
TiNi (B19′)	15	9	5
Ti_2_Ni	12	6	4

**Table 4:** Corrosion parameters obtained for all studied samples and Ti plate, TiNi plate, and thermally treated TiNi plate.

Sample	*I*_corr_, µA	*E*_corr_, mV	*R*_a_, µm	$C_{corr}^{*}$, mm/year
LP	0.936	−345.6	80	7.3 × 10^−5^
LP-20	0.533	−391.4	70	4.8 × 10^−5^
LP-30	1.44	−350.2	60	1.5 × 10^−4^
SP	0.452	−278.7	22	1.3 × 10^−4^
SP-20	1.86	−332.9	18	6.5 × 10^−4^
SP-30	0.014	−343.2	3	2.9 × 10^−5^
Ti plate	0.058	−194.9	0.48	7.6 × 10^−4^
TiNi-Pl.	0.742	−358.5	1.41	3.3 × 10^−3^
TiNi-Pl./t	0.036	−313.6	0.55	4.3 × 10^−4^

Calculated taking into account surface roughness. For the titanium plate with the lowest roughness, the roughness coefficient was taken as 1.

## Data Availability

Not applicable.

## Supplementary Materials

The following supporting information can be downloaded at: https://www.mdpi.com/article/10.3390/jfb14050277/s1, Figure S1: SEM images with different magnification for sample LP and after corrosion; Figure S2: SEM images with different magnification for sample LP-20 before and after corrosion; Figure S3: SEM images with different magnification for sample LP-30 before and after corrosion; Figure S4: SEM images with different magnification for sample SP before and after corrosion; Figure S5: SEM images with different magnification for sample SP-20 before and after corrosion; Figure S6: SEM images with different magnification for sample SP-30 before and after corrosion. Selected areas marked with red rectangles in (c) are presented in Figure S7 with larger magnification; Figure S7: SEM images of selected areas (shown in Figure S6) for sample SP-30 after corrosion; Figure S8: SEM images of materials after 3 days of cell growth; Figure S9: SEM images of materials after 3 days of cell growth; Figure S10: CLSM images of materials after 7 days of cell growth; Figure S11: CLSM images of materials after 7 days of cell growth; Figure S12: SEM images of materials after 10 days of cell growth; Figure S13: SEM images of materials after 10 days of cell growth.
