# Supplementary material for: Combined Porous-Monolithic TiNi Materials Surface-Modified with Electron Beam for New-Generation Rib Endoprostheses
Source: J Funct Biomater. 2023 May 15;14(5):277. doi: 10.3390/jfb14050277 (PMC10219054; doi:10.3390/jfb14050277)
Supplement: Supplementary file 1 [file jfb-14-00277-s001.zip › jfb-2388274-supplementary.pdf]

Supplementary materials for:

Combined Porous-Monolith TiNi Materials Surface-Modified with Electron Beam for New-Generation Rib Endoprostheses

Anastasiia V. Shabalina [1,*], Sergey G. Anikeev [1,2], Sergei A. Kulinich [3,*], Nadezhda V. Artyukhova [1], Vitaly A. Vlasov [4,*], Maria I. Kaftaranova [1], Valentina N. Hodorenko [1], Evgeny V. Yakovlev [5], Evgeny A. Pesterev [5], Anna V. Lukyanenko [6,7], Mikhail N. Volochaev [6], Sofiya Pakholkina [1], Oibek Mamazakirov [1], Victor V. Stolyarov [8], Anatolii V. Mokshin [2], Victor E. Gunther [1]

[1] Laboratory of Medical Materials Science, Tomsk State University, 634050 Tomsk, Russia
[2] Institute of Physics, Kazan Federal University, 420008 Kazan, Russia
[3] Research Institute of Science and Technology, Tokai University, Hiratsuka-shi, 259-1292 Kanagawa, Japan
[4] Research School of High-Energy Physics, National Research Tomsk Polytechnic University, 634050 Tomsk, Russia
[5] Tomsk Scientific Center, Siberian Branch of Russian Academy of Sciences, 634055 Tomsk, Russia
[6] Kirensky Institute of Physics Federal Research Center KSC Siberian Branch Russian Academy of Science, 660036 Krasnoyarsk, Russia
[7] Siberian Federal University, 660041 Krasnoyarsk, Russia
[8] Department of Morphology and Physiology of the Medical Institute, Surgut State University, 628403 Surgut, Russia

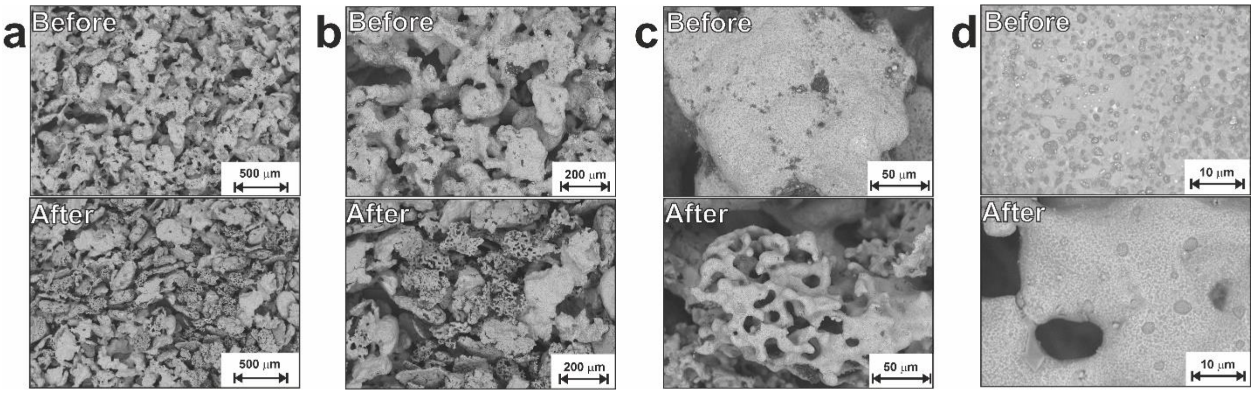

Figure S1. SEM images with different magnification for sample LP before (upper line) and after (lower line) corrosion.

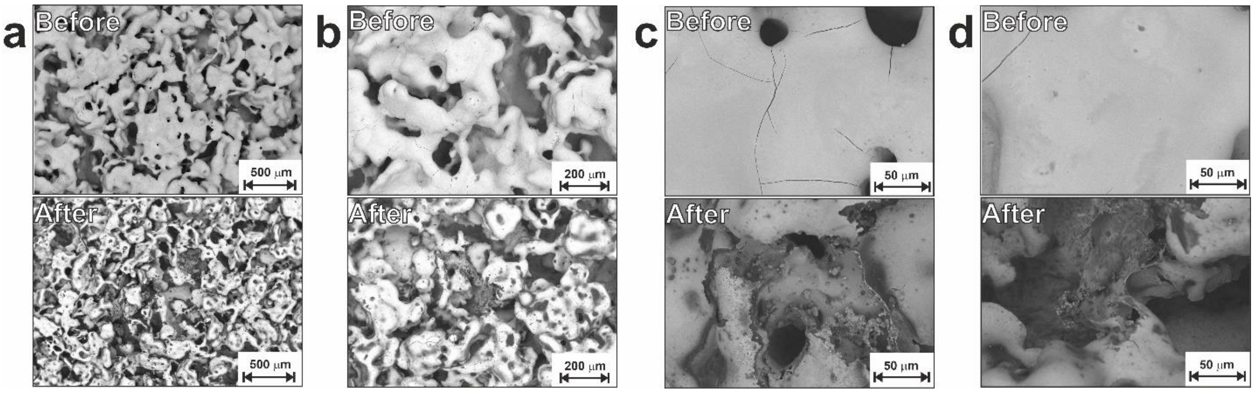

Figure S2. SEM images with different magnification for sample LP-20 before (upper line) and after (lower line) corrosion.

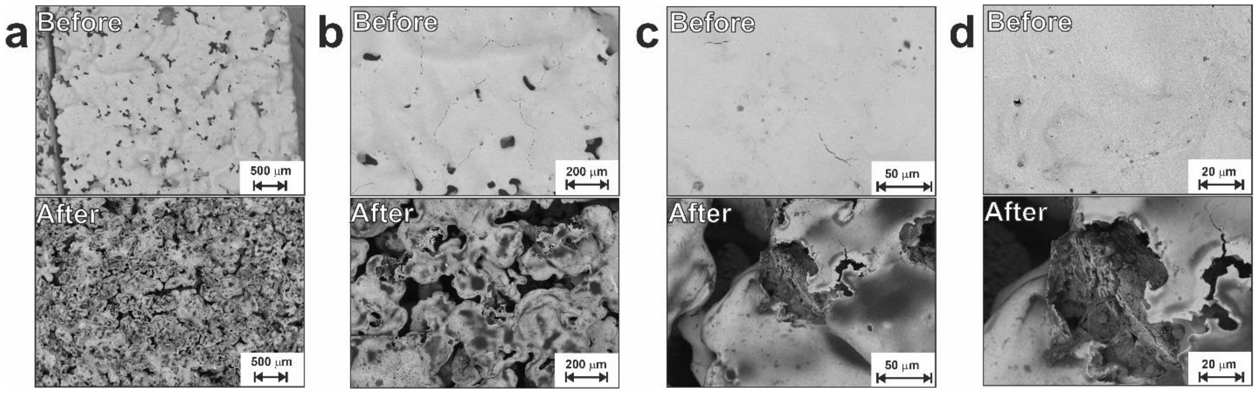

Figure S3. SEM images with different magnification for sample LP-30 before (upper line) and after (lower line) corrosion.

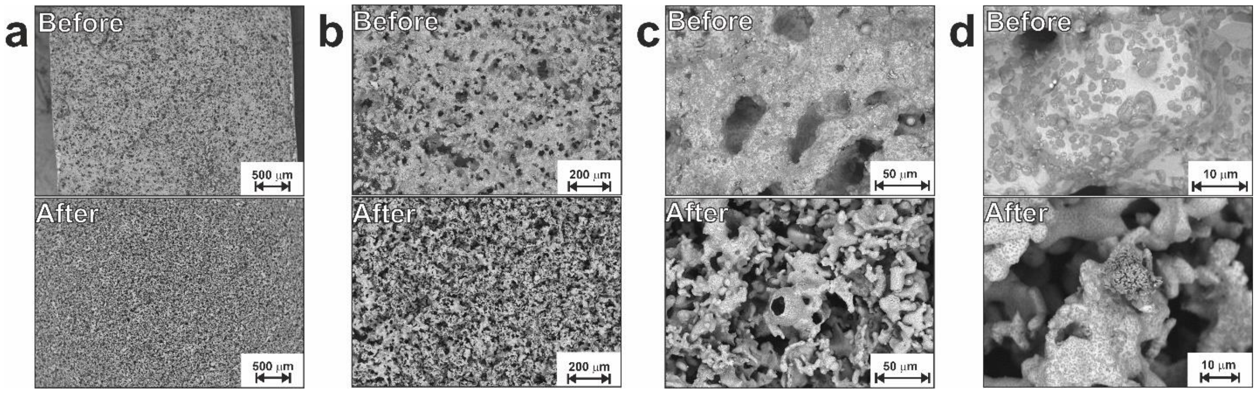

Figure S4. SEM images with different magnification for sample SP before (upper line) and after (lower line) corrosion.

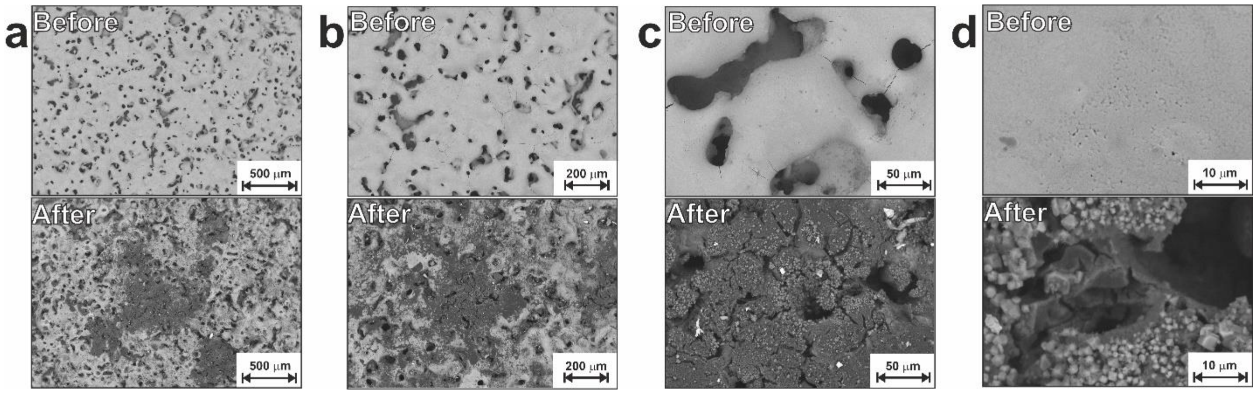

Figure S5. SEM images with different magnification for sample SP-20 before (upper line) and after (lower line) corrosion.

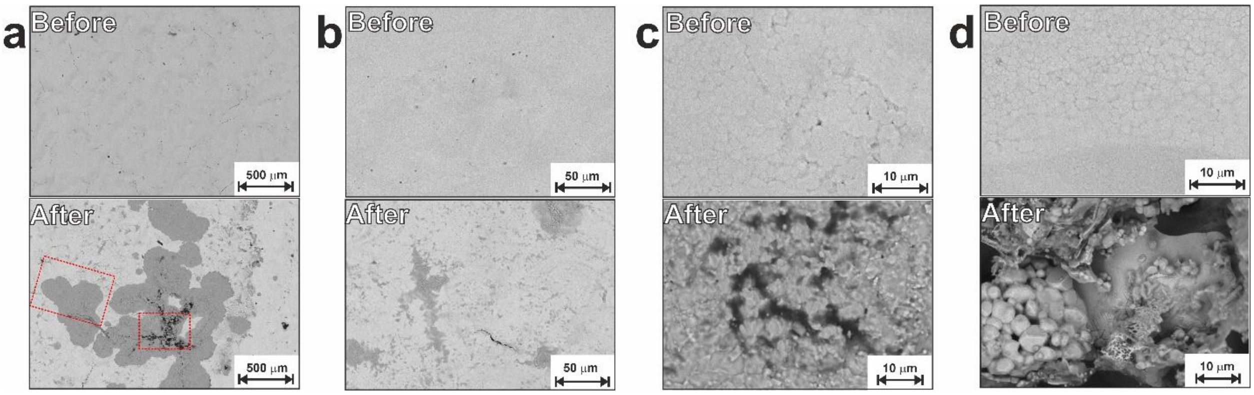

Figure S6. SEM images with different magnification for sample SP-30 before (upper line) and after (lower line) corrosion. Selected areas marked with red rectangles in (c) are presented in Fig. S7 with larger magnification.

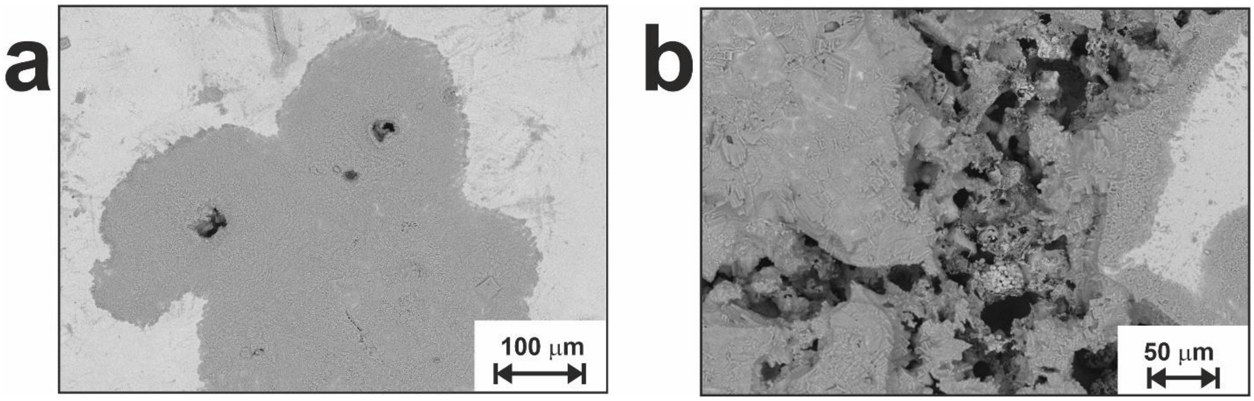

Figure S7. SEM images of selected areas (shown in Fig.S6) for sample SP-30 after corrosion.

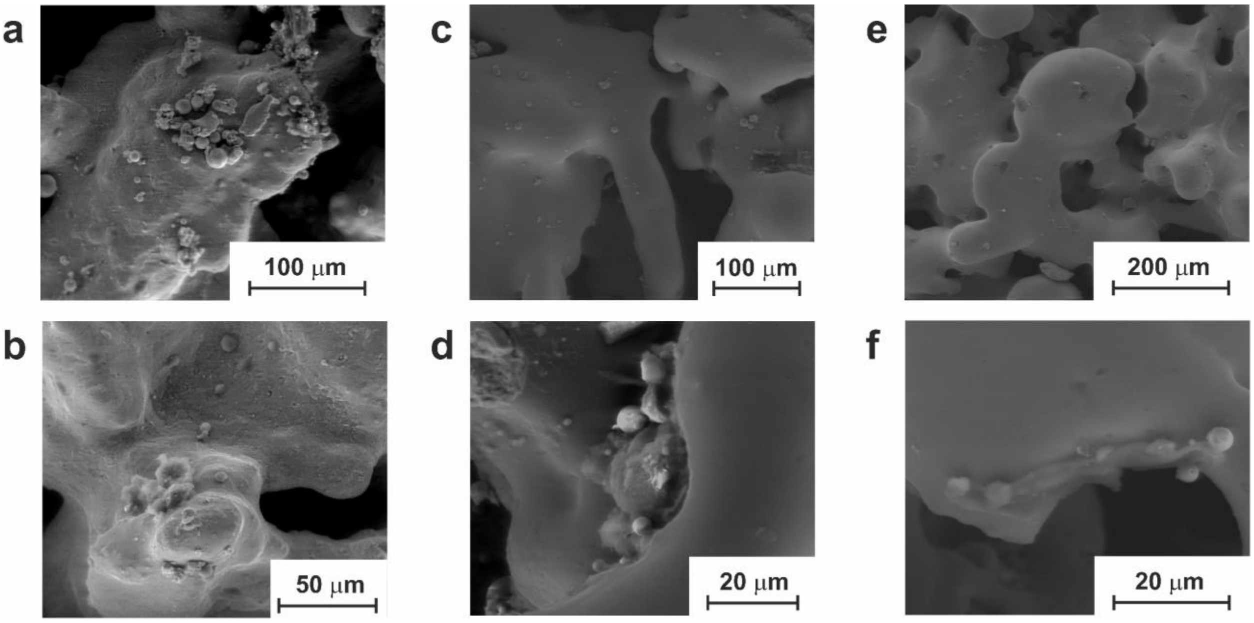

Figure S8. SEM images of materials after 3 days of cell growth: (a, b) LP; (c, d) LP-20; (e, f) LP-30.

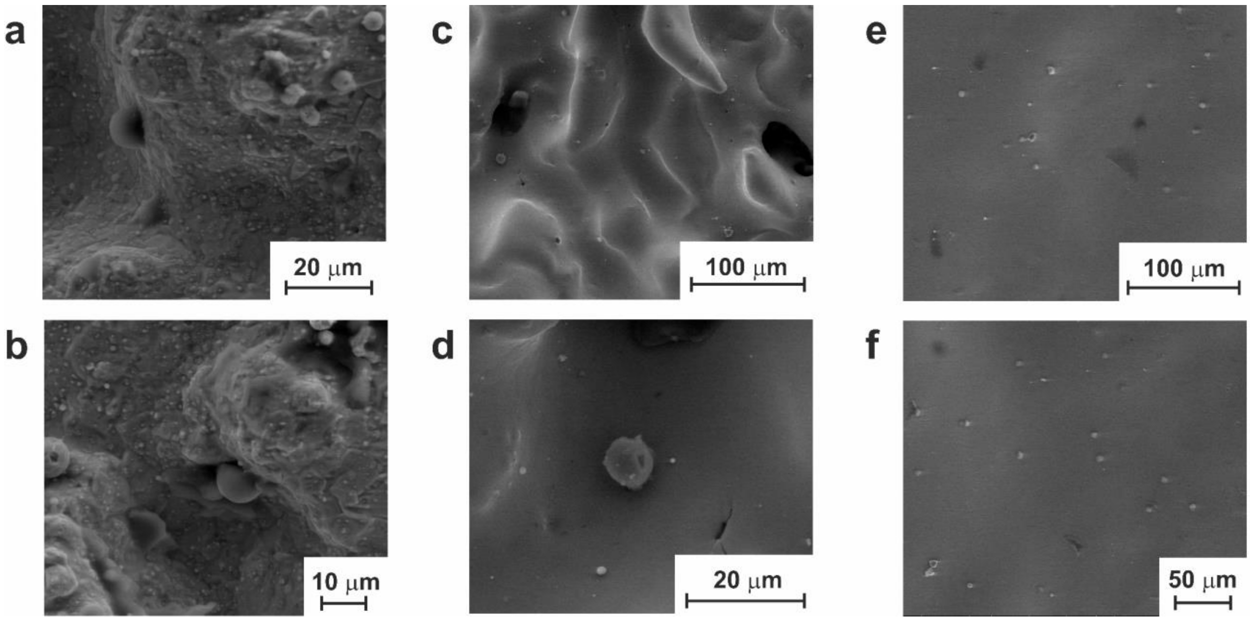

Figure S9. SEM images of materials after 3 days of cell growth: (a, b) SP; (c, d) SP-20; (e, f) SP-30.

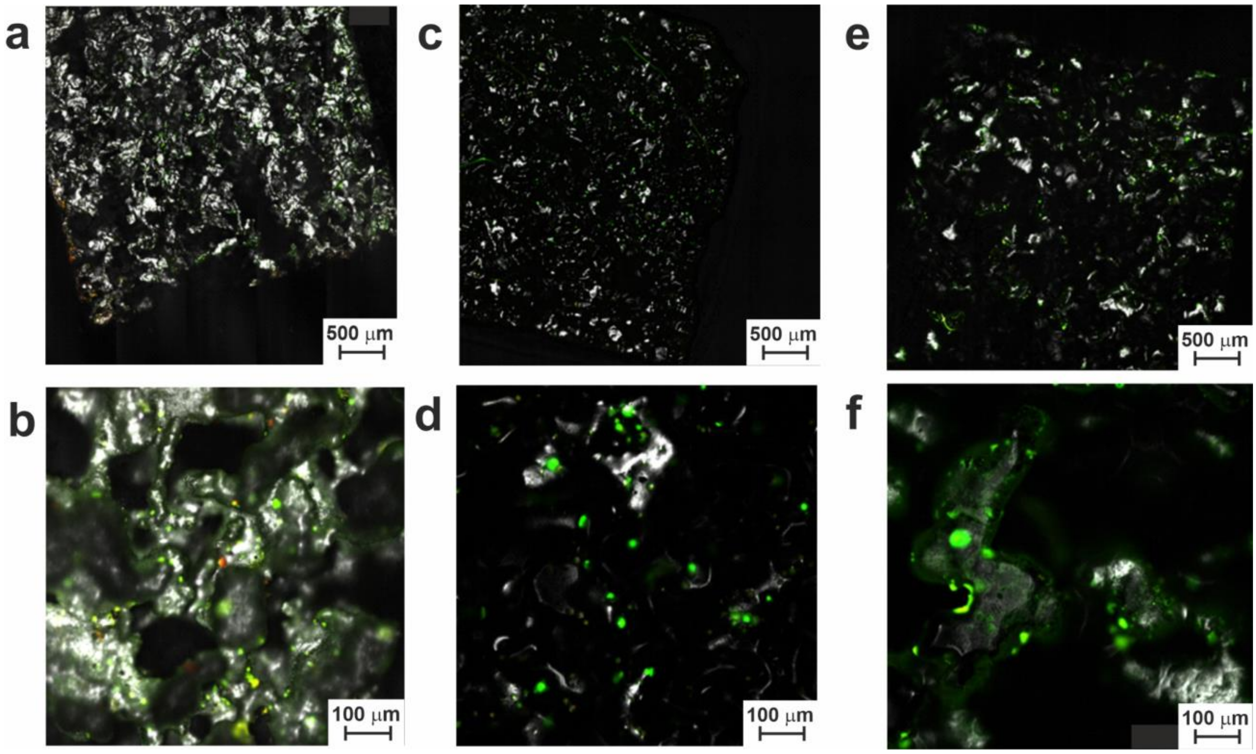

Figure S10. CLSM images of materials after 7 days of cell growth: (a, b) LP; (c, d) LP-20; (e, f) LP-30.

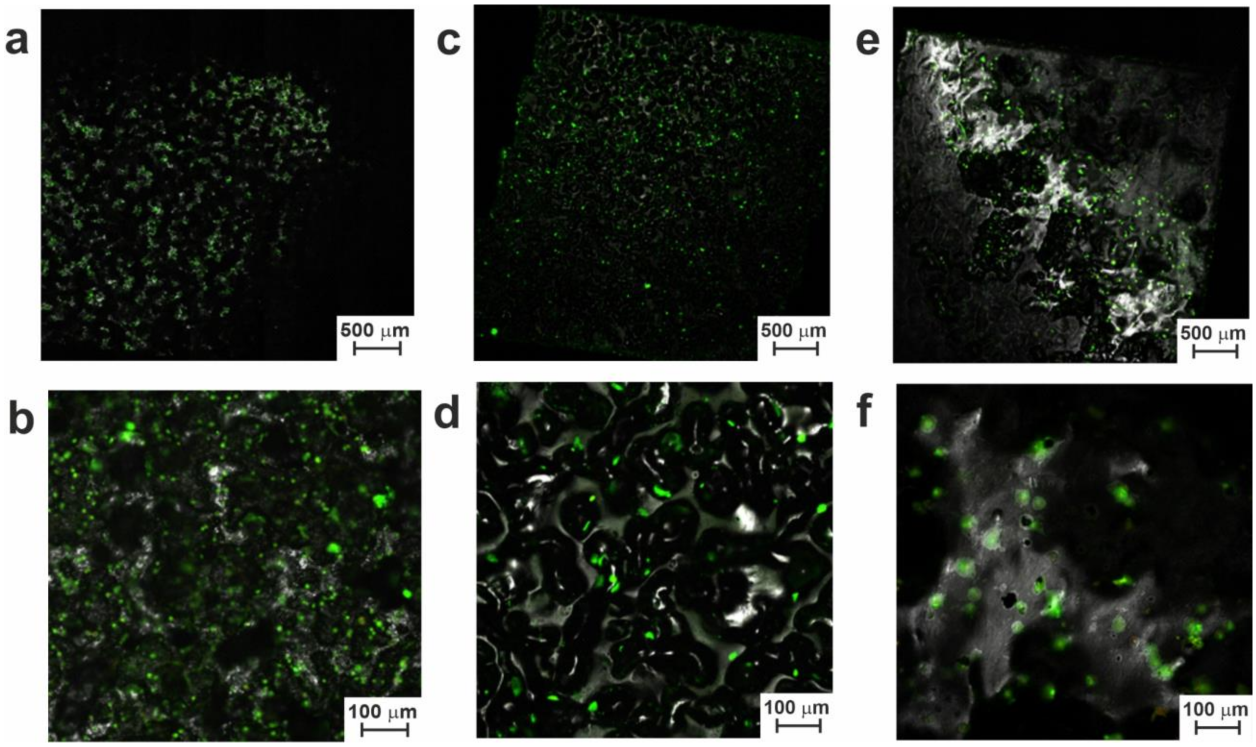

Figure S11. CLSM images of materials after 7 days of cell growth: (a, b) SP; (c, d) SP-20; (e, f) SP-30.

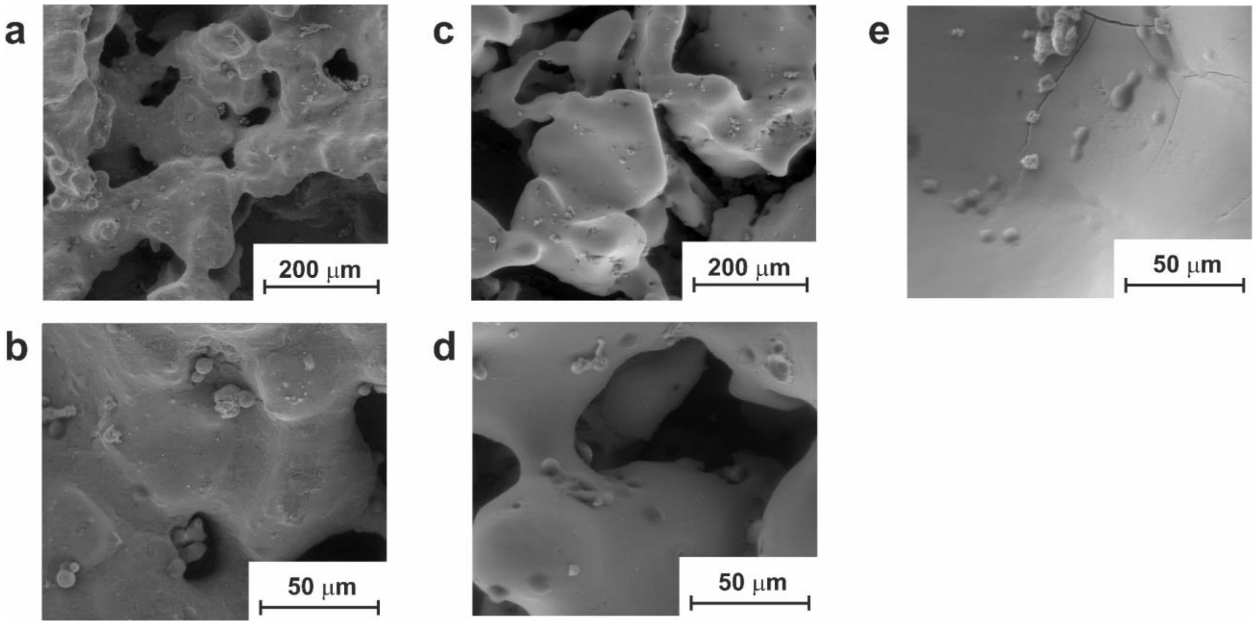

Figure S12. SEM images of materials after 10 days of cell growth: (a, b) LP; (c, d) LP-20; (e, f) LP-30.

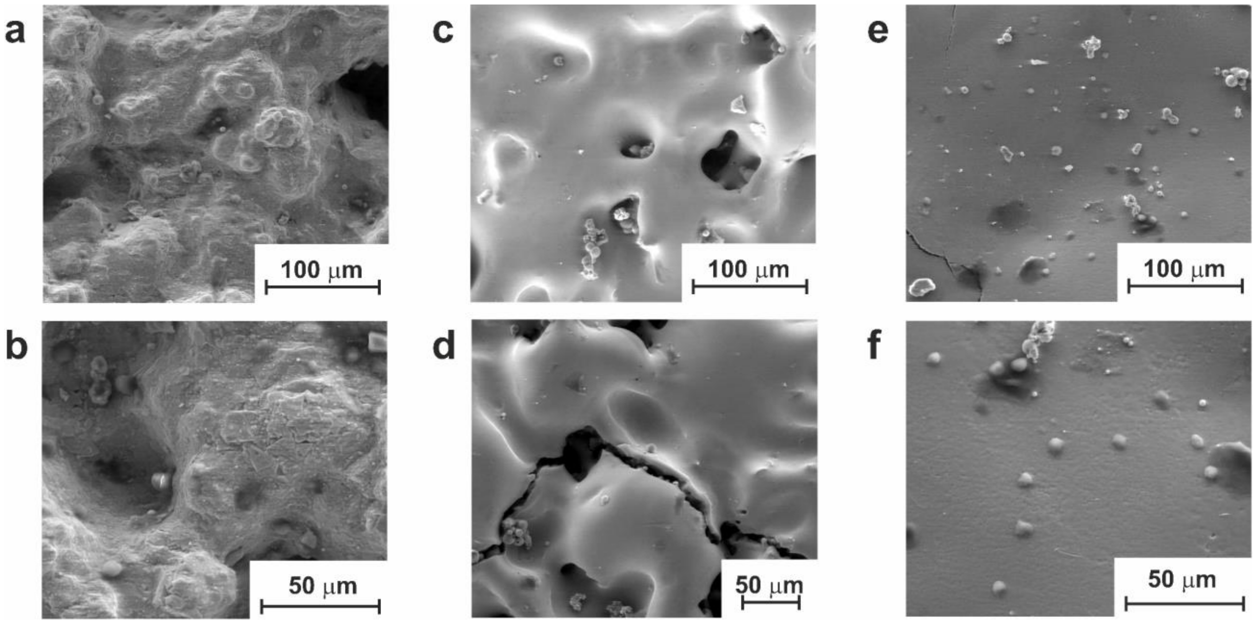

Figure S13. SEM images of materials after 10 days of cell growth: (a, b) SP; (c, d) SP-20; (e, f) SP-30.
